# Pentoxifylline Alleviates Cardiac Ischemia and Dysfunction Following Experimental Angina in Insulin Resistance

**DOI:** 10.1371/journal.pone.0098281

**Published:** 2014-05-29

**Authors:** Ahmad Azhar, Hany M. El-Bassossy

**Affiliations:** 1 Department of Pediatrics, Faculty of Medicine, King Abdulaziz University, Jeddah, Kingdom of Saudi Arabia; 2 Department of Pharmacology, Faculty of Pharmacy, King Abdulaziz University, Jeddah, Kingdom of Saudi Arabia; 3 Department of Pharmacology, Faculty of Pharmacy, Zagazig University, Zagazig, Egypt; Georgia Regents University, United States of America

## Abstract

We have previously shown that pentoxifylline (PTX) protects from vascular complications associated with insulin resistance (IR). Here, we investigated the protective effect of PTX against cardiac ischemia and dysfunction following experimental angina in IR. IR, along with its accompanying cardiac dysfunction, was induced in rats by a high-fructose (10% in drinking water) high-fat diet for 12 weeks. PTX was administered daily (30 mg⋅kg^−1^) during the last 4 weeks of the study. Experimental angina was induced by isoproterenol (10 µg⋅kg^−1^) administered by intravenous injection. Both before (baseline) and after the experimental angina, cardiac contractility was assessed by continuous recording in anesthetized rats via a microtip catheter inserted in the left ventricle, and cardiac conductivity was determined by a surface electrocardiograph. Serum glucose, insulin, tumor necrosis factor-α (TNFα), and adiponectin levels and lipid profile were also determined. Feeding the rats a high-fructose high-fat diet produced IR, as evidenced by significant hyperinsulinemia and hyperglycemia, and PTX administration did not affect this IR. When subjected to experimental angina, IR hearts were less resistant to the ischemia following induction of angina (reflected by the large ST height depression) compared with controls, and PTX completely prevented the excessive ST height depression in IR animals. In addition, left ventricular pressure development was largely attenuated during and after induction of angina in IR animals compared with controls. PTX administration prevented the excessive attenuation in ventricular pressure development in IR animals. IR was associated with elevated levels of the inflammatory cytokine TNFα, whereas PTX treatment elevated the serum level of the anti-inflammatory cytokine adiponectin. PTX alleviates cardiac ischemia and dysfunction following experimental angina in IR directly through inhibition of the low-grade inflammation that accompanies IR.

## Introduction

Insulin resistance (IR) is a major and growing healthcare problem throughout the world and is a key component of the metabolic syndrome, which represents a major risk factor for cardiovascular diseases development [1]. IR is a component of glucose intolerance [2], that is compensated by prolonged hyperinsulinemia. Eventually, the failure of pancreatic β-cells to secrete sufficient insulin, leading to type II diabetes mellitus [3].

Cardiovascular disorders continue to constitute a major cause of mortality and morbidity in diabetic patients despite the significant achievements in diabetes diagnosis and treatment [4]. Angina pectoris, caused by an imbalance between the supply and demand for oxygen in the heart, is divided into 2 types depending on its cause: (1) angina of effort and (2) stable angina. The former is a myocardial ischemia induced by a lack of oxygen, which is caused by an inability to increase blood flow due to coronary constriction while stable angina occurs due to coronary vasospasm during rest [5]. Electrocardiography (ECG) is an important diagnostic tool for angina pectoris because myocardial ischemia is closely reflected by elevation or depression in the ST segment [6]. ST segment elevation has been also reported in some disease states such as acute pericarditis and Brugada syndrome [7]. However, these disease conditions are completely distinguished from the isoproterenol-induced angina model used in the present study.

In the past decade, it has been increasingly recognized that IR and its associated vascular complications are accompanied by chronic, low-grade systemic inflammation [8]. Pentoxifylline (PTX) is a phosphodiesterase inhibitor that is widely used in peripheral vascular disease treatment and for which a wide range of reported immunomodulatory activities have been described [9].

The aim of the present study was to investigate the potentially protective effect of PTX against cardiac ischemia and dysfunction following experimental angina in IR and elucidate the mechanism of this protective effect.

## Materials and Methods

### Ethics Statement

Our studies were carried out in strict accordance with institutional animal care and use guidelines. Animals were housed in constant light/dark cycles with access to a standard rodent diet *ad libitum*. The experimental protocol was approved (Reference No 1320-13) by the Unit of Biomedical Ethics Research Committee, King Abdulaziz University.

### Study protocol

Six-week-old male Wistar rats (King Abdulaziz University) were randomly divided into 4 experimental groups (8 animals each): control, IR, PTX-treated IR, and PTX-treated control. IR was induced by adding fructose (10%) to the drinking water and feeding rats a high-fat high-salt diet containing 16% crude protein, 28.2% crude fat, 2.8% crude fiber, 4.8% ash, and 3.4% salt for 12 weeks. Control animals received tap water and a standard diet containing 20% crude protein, 4% crude fat, 3.5% crude fiber, 6% ash, and 0.5% salt. PTX (30 mg⋅kg^−1^⋅day^−1^) [10] was administered daily during the last 4 weeks by dissolving it in drinking water (140–160 mg⋅L^−1^ depending upon water consumption) [11]. The amount of water consumed was measured, and the PTX concentration in the drinking water was readjusted weekly.

At the end of the study, animals were fasted for 8 hours, after which, fasting blood glucose level was determined. Rats were then anesthetized by an intraperitoneal injection of urethane (1.5 g⋅kg^−1^) [12]. Venous blood (1 mL) was withdrawn, allowed to coagulate for 30 min at 4°C, and then centrifuged (3000×*g* at 4°C for 20 min) to separate serum. The serum was divided into aliquots and stored at −20°C until analysis for insulin, tumor necrosis factor-α (TNFα), adiponectin, and lipid profile. Rats were injected with 1 mL saline to prevent hypovolemia. A microtip catheter was then inserted into the left ventricle through an opening in the right carotid artery to continuously record cardiac contractility while cardiac conductivity was determined by a surface ECG. After 15 min of recording basal cardiac contractility and conductivity, experimental angina was induced in all animal groups by isoproterenol (10 µg⋅kg^−1^) injection through the femoral vein [13], after which, cardiac contractility and conductivity were continuously recorded for another 30 min. The effect of induced angina on cardiac contractility and conductivity were compared between different groups.

### Biochemical analysis

Glucose was determined in tail blood by a glucose meter (Bionime GmBH) using noble metal electrode strips. The serum insulin level was measured by enzyme-linked immunosorbent assay (ELISA) (Millipore, Billerica, MA, USA) that uses a plate coated with monoclonal anti-rat insulin antibodies. Serum TNFα and adiponectin levels were determined by ELISA using the Quantikine kit (R&D Systems, Minneapolis, MN, USA) using rat TNFα or rat adiponectin and antibodies raised against rat TNFα or rat adiponectin, respectively. Serum triglyceride, total cholesterol, and high-density lipoprotein (HDL)-cholesterol levels were determined using the ELITech assay kit (ELITech; Laindon, Essex, France).

### Cardiac contractility recording

Invasive real-time recording of cardiac contractility was carried out according to the method described by Radovits et al [14] with slight modifications. After urethane anaethesia, animals were placed on controlled heating pads, and body temperature, measured via a rectal probe, was maintained at 37°C. A microtip pressure transducer (SPR-320; Millar Instruments, Houston, TX, USA) was inserted into the right carotid artery and advanced into the left ventricle under pressure control. After stabilization for 5 min, the signals were continuously recorded at a sampling rate of 1000 s^−1^. The microtip catheter was connected to a PowerLab Data Interface Module connected to a PC running LabChart professional software (version 7.3; ADI Instruments, Bella Vista, Australia) containing a blood pressure module, which detects and calculates the slopes of the systolic pressure increment (dP/dt) and the diastolic pressure decrement (−dP/dt), the systolic and diastolic duration, and the contractility index.

### Cardiac conductivity recording

The standard limb lead II of the surface ECG was recorded by the Powerlab system (ADI Instruments) connected to a PC running LabChart professional software (version 7.3) containing an ECG module, which detects different components of the ECG. The change in the ST segment height was used as an index of angina severity. The mean ECG voltage 13 ms after the peak of the S wave was defined as the value of the ST segment, as described previously [15]. The difference in amplitude of the ST segment before and after the administration of the isoproterenol was calculated and expressed as the ST segment depression in millivolts.

### Drugs and chemicals

The following drugs and chemicals were used: Pentoxifylline, isopreterenol, urethane (Sigma-Aldrich, St. Louis, MO, USA). Pentoxifylline was dissolved in distilled water while urethane and isoproterenol were dissolved in saline.

### Statistical analysis

Values are expressed as mean ± SEM. Statistical analysis was performed by analysis of variance (ANOVA) followed by Newman-Keuls post hoc test using Prism software (version 5; GraphPad, San Diego, CA, USA).

## Results

### IR parameters

The high-fructose high-fat diet for 12 weeks produced IR as indicated by the significant elevation in blood glucose (p<0.001), fructosamine (p<0.01), and insulin (p<0.01) levels compared with control animals. PTX administration did not significantly affect the hyperglycemia or hyperinsulinemia associated with this diet. PTX administration to normal animals did not significantly affect the blood glucose, fructosamine, or insulin levels (Table 1).

**Table 1:** Effect of daily oral administration of pentoxifylline (PTX, 30 mg⋅kg^−1^, last 4 weeks) on high fructose high fat diet- induced insulin resistance (IR, 10% fructose in drinking water plus 25% unsaturated fat in diet, for 12 weeks) associated changes in serum levels of fasting glucose, fructosamine and insulin.

Treatment	Glucose	Fructosamine	Insulin
	(mg.dl^−1^)	(mg.dl^−1^)	(µg.l^−1^)
**C**	71.9±2.9	46.63±3.8	0.8±0.1
**IR**	108.7±4.2^***^	71.5±6.1^**^	3.2±0.3^**^
**IR-PTX**	111.4±6.8	66.8±3.9	3.8±0.7
**C-PTX**	95.0±3.9	62.7±1.6	1.0±0.1

Values are expressed as the mean ± S.E of mean; N = 6–8 animals; ^**^P<0.01, ^***^P<0.001, compared with the corresponding control group values; by One Way ANOVA and Newman Keuls *post hoc* test.

### Isoproterenol-induced angina

#### Effect on cardiac conductivity

**Figure 1:**
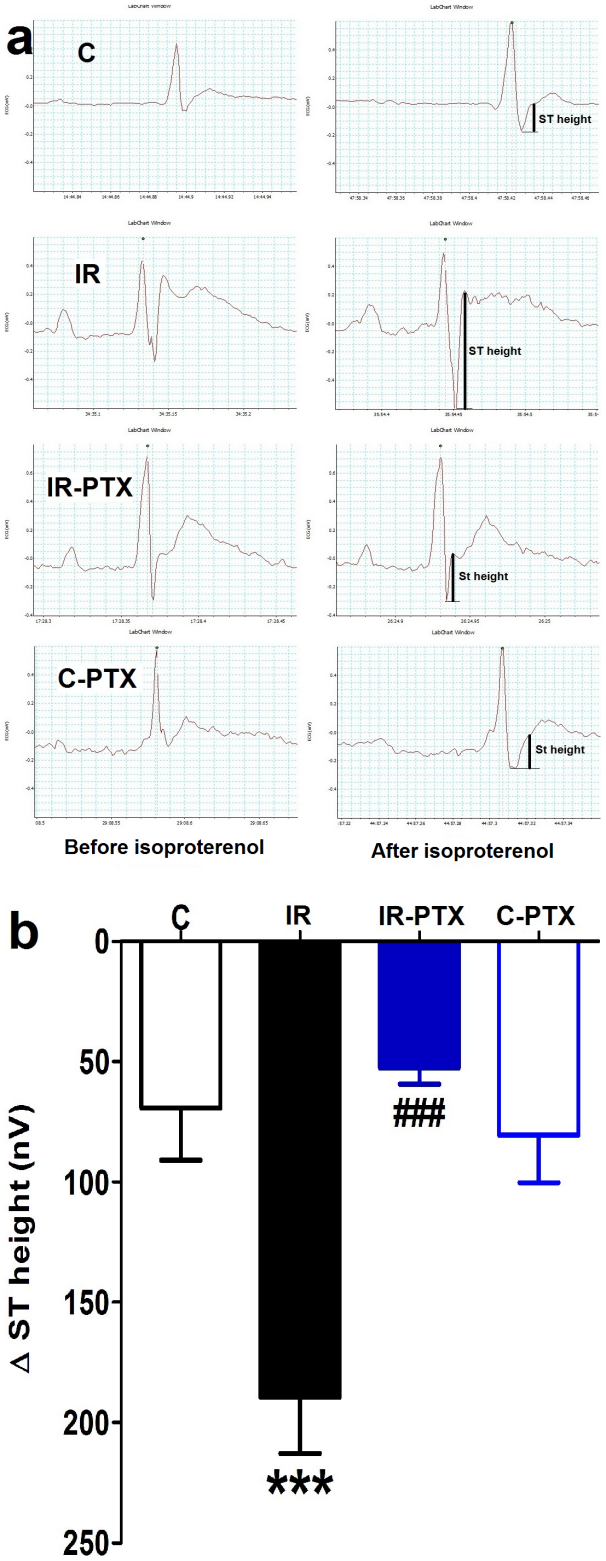
Effect of pentoxifylline (PTX) on ECG and ST height depression following isoproterenol injection. Insulin resistance (IR) was induced in rats with a high-fat high-fructose diet fed for 12 weeks, and PTX (30 mg⋅kg^−1^⋅day^−1^) was administered during the last 4 weeks of the study in IR-PTX and C-PTX groups while control (C) received normal diet. The recorded ECG before and after isoproterenol injection in all groups (a) and isoproterenol-induced ST height depression (b) is shown. By one-away ANOVA and Newman Keuls post hoc test: ***p<0.001, compared with the corresponding control group; ^###^p<0.001 compared with the corresponding IR group.

Intravenous injection of isoproterenol led to induction of cardiac angina in anaesthetized rats as indicated by ST height depression. Animals with IR were less resistant to an anginal attack and characterized by more severe cardiac ischemia as indicated by the significantly larger depression in ST height (p<0.01) compared with control. PTX administration significantly alleviated the ischemia in the hearts of IR animals as indicated by the significant decrease in ST height depression compared with the untreated IR group (all at p<0.05). PTX administration to normal animals did not significantly affect the cardiac ischemia following the anginal attacks (Figure 1).

#### Effect on cardiac contractility

**Figure 2:**
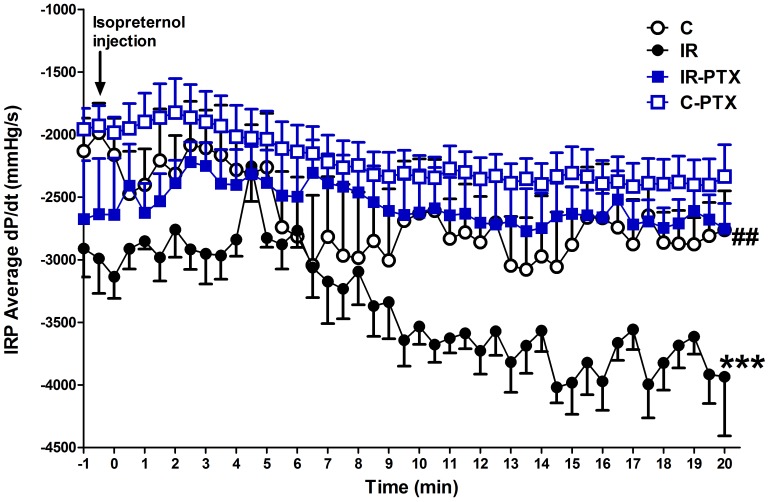
Effect of pentoxifylline (PTX) on left ventricular pressure development. Insulin resistance (IR) was induced in rats with a high-fat high-fructose diet fed for 12 weeks, and PTX (30 mg⋅kg^−1^⋅day^−1^) was administered during the last 4 weeks of the study in IR-PTX and C-PTX groups while control (C) received normal diet. The recorded average left ventricular pressure development (dP/dt) after isoproterenol injection in all groups is shown. By one-away ANOVA and Newman Keuls post hoc test: ***P<0.001, compared with the corresponding control group; ^##^P<0.01, compared with the corresponding IR group.

Following isoproterenol injection, the left ventricular pressure development was attenuated. Animals with IR were characterized by greater attenuation of the left ventricular pressure development as indicated by the significant decrease in average dP/dt (p<0.001) compared with control animals. PTX administration completely blocked the excessive decrease in average dP/dt (p<0.01) compared with untreated IR animals. PTX administration to normal animals did not significantly affect the left ventricular pressure development following anginal attacks (Figure 2).

**Figure 3:**
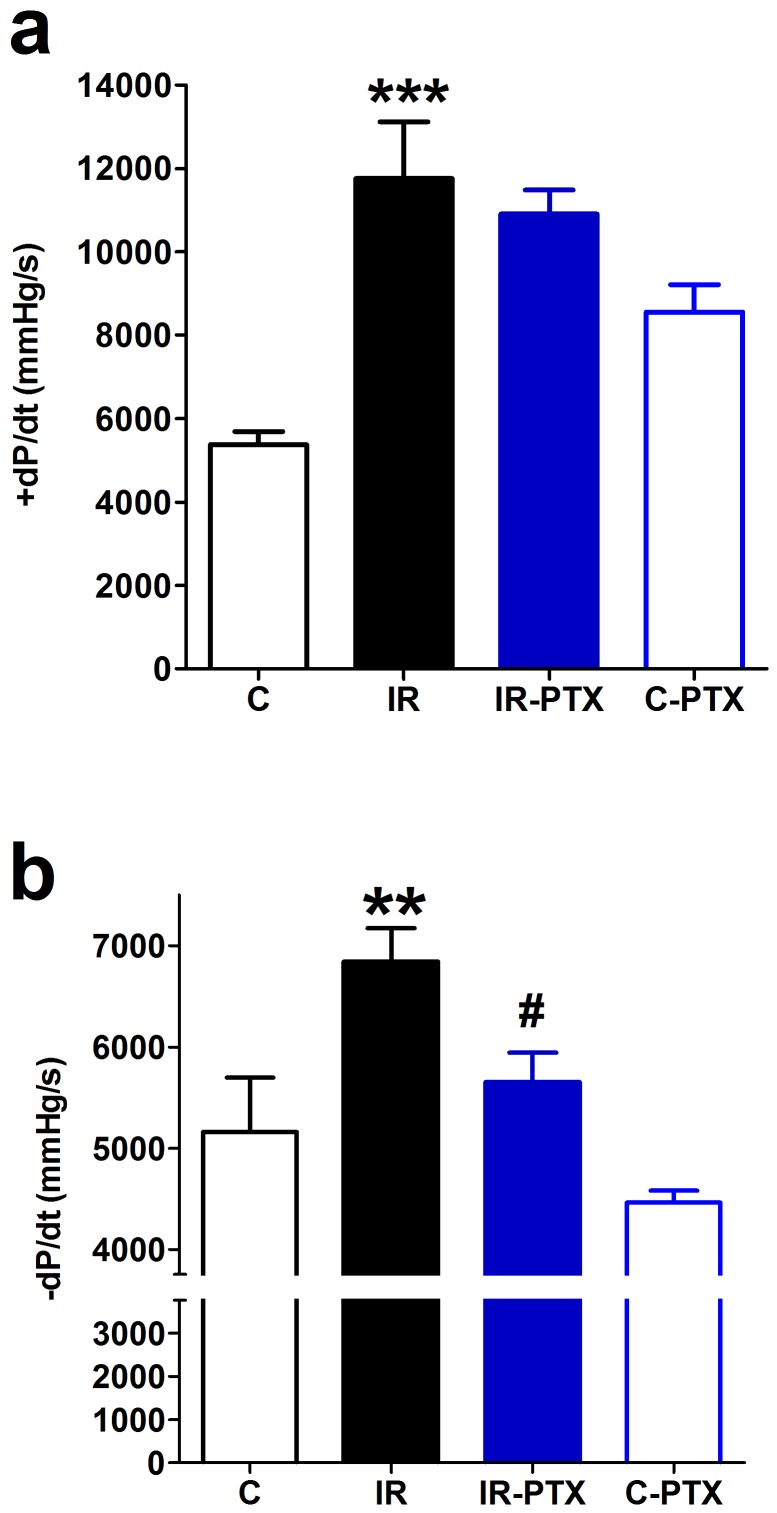
Effect of pentoxifylline (PTX) on left ventricular pressure increment and decrement. Insulin resistance (IR) was induced in rats with a high-fat high-fructose diet fed for 12 weeks, and PTX (30 mg⋅kg^−1^⋅day^−1^) was administered during the last 4 weeks of the study in IR-PTX and C-PTX groups while control (C) received normal diet. The recorded left ventricular pressure increment (a) and decrement (b) following isoproterenol injection in all groups is shown. By one-away ANOVA and Newman Keuls post hoc test: **P<0.01, ***P<0.001, compared with the corresponding control group; #P<0.05 compared with the corresponding IR group.

Following induction of angina by isoproterenol, IR animals showed greater cardiac systolic dysfunction as indicated by the significant increase in the slope of the systolic pressure increment (p<0.001) compared with controls. However, PTX administration did not have any significant effect on the systolic dysfunction in IR animals (Figure 3a). However, compared with controls, IR animals showed a marked cardiac diastolic dysfunction as indicated by the significant decrease in the slope of the diastolic pressure decrement (p<0.01), which was significantly alleviated by PTX administration (p<0.05) (Figure 3b). PTX administered to normal animals affected neither the slope of the systolic pressure increment nor the slope of the diastolic pressure decrement following anginal attacks (Figure 3).

IR animals did not show significant changes in the cardiac contractility index, cycle duration, or systolic or diastolic duration compared with control animals, and PTX administration did not affect any of these parameters in IR or normal animals (Table 2).

**Table 2:** Effect of daily oral administration of pentoxifylline (PTX, 30 mg⋅kg^−1^, last 4 weeks) on high fructose high fat diet- induced insulin resistance (IR, 10% fructose in drinking water plus 25% unsaturated fat in diet, for 12 weeks) associated changes in left ventricle contractility index, cycle duration, systolic duration and diastolic duration.

Treatment	Contractility index	Cycle duration	Systolic duration	Diastolic duration
	(1/s)	(ms)	(ms)	(ms)
**C**	128.9±14.5	209.1±19.1	81.9±7.1	127.2±12.3
**IR**	160.3±8.0	186.2±5.1	83.9±2.2	102.2±3.5
**IR-PTX**	189.0±11.2	209.9±5.4	90.1±2.9	119.8±5.0
**C-PTX**	171.6±9.2	206.7±4.4	92.7±2.4	114.0±2.7

Values are expressed as the mean ± S.E of mean; N = 6–8 animals; No statistical difference has been detected by One Way ANOVA and Newman Keuls *post hoc* test.

### Low-grade inflammation

**Figure 4:**
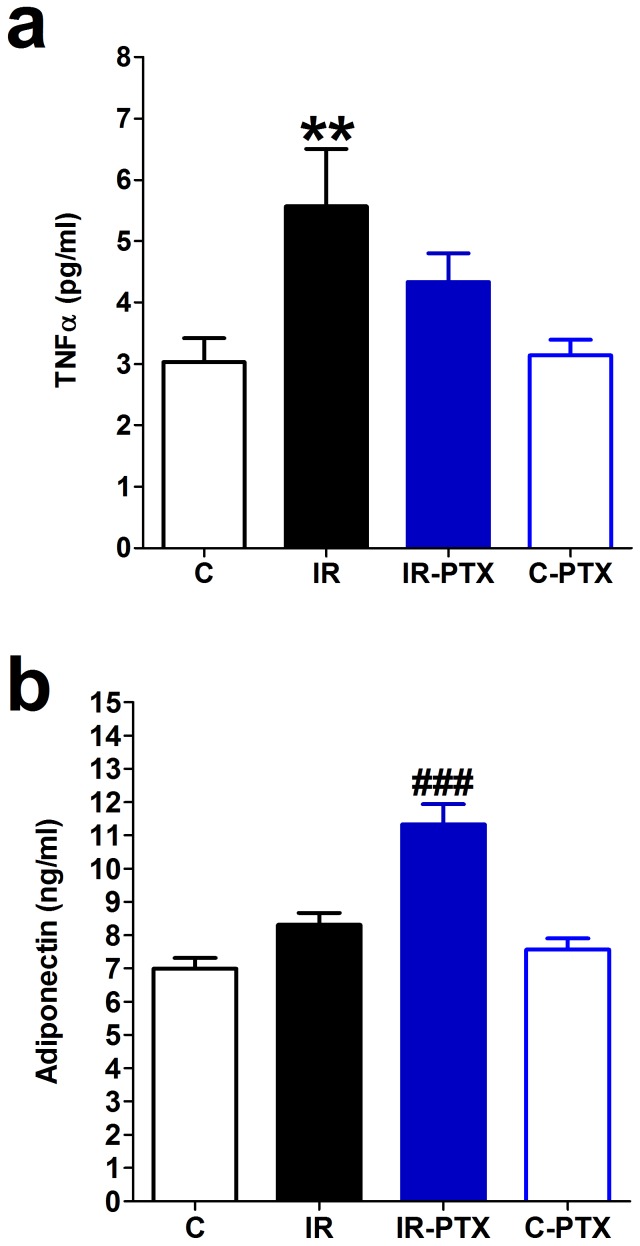
Effect of pentoxifylline (PTX) on serum tumor necrosis factor-α (TNFα) and adiponectin levels. Insulin resistance (IR) was induced in rats with a high-fat high-fructose diet fed for 12 weeks, and PTX (30 mg⋅kg^−1^⋅day^−1^) was administered during the last 4 weeks of the study in IR-PTX and C-PTX groups while control (C) received normal diet. The effect different treatments on the serum TNFα (a) and adiponectin (b) levels is shown. By one-away ANOVA and Newman Keuls post hoc test: **P<0.01, compared with the corresponding control group; ###P<0.001 compared with the corresponding IR group.

IR induced by a high-fructose high-fat diet was associated with low-grade inflammation, reflected by a significant increase in serum levels of the inflammatory cytokine TNFα (p<0.01, Figure 4a) compared with controls. However, the serum levels of the anti-inflammatory cytokine adiponectin was not significantly changed in the IR animals compared with controls (Figure 4b). PTX administration alleviated the low-grade inflammation associated with IR as indicated by the significant increase in serum adiponectin levels (p<0.001) and a tendency toward a decrease in TNFα compared with the untreated IR group. PTX administered to normal animals did not significantly affect the serum TNFα or adiponectin levels (Figure 4).

### Serum lipids

The IR model used in this study did not show significant changes compared with controls in the serum triglyceride, cholesterol, or HDL-cholesterol levels, which were also not affected by PTX administration in normal or IR animals (Table 3).

**Table 3:** Effect of daily oral administration of pentoxifylline (PTX, 30 mg⋅kg^−1^, last 4 weeks) on high fructose high fat diet- induced insulin resistance (IR, 10% fructose in drinking water plus 25% unsaturated fat in diet, for 12 weeks) associated changes in serum levels of triglycerides, total cholesterol and HDL-cholesterol.

Treatment	Triglycerides	Total cholesterol	HDL-Cholesterol
	(mg.dl^−1^)	(mg.dl^−1^)	(mg.dl^−1^)
**C**	47.3±2.7	48.5±8.1	13.9±2.9
**IR**	62.6±14.7	48.6±4.3	22.5±2.8
**IR-PTX**	69.8±7.2	58.5±3.6	22.5±1.8
**C-PTX**	41.8±5.3	45.4±11.5	18.6±4.6

Values are expressed as the mean ± S.E of mean; N = 6–8 animals; No statistical difference has been detected by One Way ANOVA and Newman Keuls *post hoc* test.

## Discussion

The current study is, to the best of our knowledge, the first to report on the potential protective effect of PTX against the cardiac ischemia and dysfunction that follows experimental angina in IR. PTX inhibited the development of cardiac ischemia and dysfunction in high-fructose high-fat diet-induced IR in rats. The following findings can explain the ability of PTX to counteract cardiac ischemia and dysfunction accompanying IR: (1) PTX alleviated the exaggerated cardiac ischemia following experimental angina in IR, (2) the attenuated left ventricular pressure development in IR was alleviated by PTX, and (3) PTX alleviated the low-grade inflammation associated with IR. These findings provide convincing evidence that PTX offsets the cardiac ischemia and dysfunction following experimental angina accompanying IR through inhibition of the low-grade inflammation.

Our results show that animals with IR were less resistant to anginal attack and characterized by more severe cardiac ischemia as indicated by the significantly larger depression in ST height compared with controls. It has been widely agreed that in diabetes and IR, the heart is less resistant to ischemia during anginal attacks. In addition, the metabolic syndrome is associated with a high incidence of coronary atheroma and an increased risk of a fatal coronary event [16]. In the present study, PTX administration significantly alleviated the ischemia induced in the hearts of IR animals as indicated by the significant decrease in ST height depression compared with the untreated IR group. This is in accordance with the cardioprotective effect of PTX against ischemia-reperfusion injury seen in isolated rat hearts [17].

Ischemia is always followed by myocardial contractile dysfunction [18]. In the present study, we found that the left ventricular pressure development was attenuated during and after induction of angina. Animals with IR were characterized by greater attenuation in left ventricular pressure development as indicated by the significant decrease in average dP/dt compared with controls. However, PTX administration completely blocked the excessive decrease in average dP/dt compared with untreated IR animals. This is in accordance with the reported inotropic effect of PTX in cardiac hypertrophy [19]. In addition, PTX restored the depressed cardiac performance after trauma-hemorrhage and resuscitation [20].

The IR animals in our study showed greater cardiac systolic and diastolic dysfunction following induction of angina by isoproterenol compared with controls. However, PTX administration protected against the cardiac diastolic dysfunction only.

The left ventricular diastolic dysfunction has been shown in the present study and indicated in previous work [21], [22]. Ventricular relaxation is an ATP-dependent active process. It depends mainly on Ca^2+^ uptake by the sarcoplasmic reticulum during the diastole. End-diastolic stiffness is largely affected by the alterations in myocardial components [23]. The protective effect of PTX seen in the present study is in accord with previous work that related the cardioprotective effect of PTX to its role on decreased intracellular Ca^2+^ overload [24].

Because PTX did not have a significant effect on the IR parameters but did significantly alleviate the cardiac ischemia and dysfunction with IR, the protective effect of PTX against cardiac ischemia and dysfunction in animals with IR seems to be a direct cardiovascular protective effect rather than a direct effect on the IR itself.

The mechanisms by which PTX protects against cardiac ischemia and dysfunction in animals with IR could be mediated by reduction of the associated low-grade inflammation. Increasing evidence indicates that a cytokine-induced low-grade inflammation is involved in the pathogenesis of diabetic complications [25]. We previously found elevated levels of the inflammatory cytokine TNFα in animal models of IR that were significantly correlated with vascular complications [26], [27]. In the present study, animals with IR were characterized by marked low-grade inflammation as evidenced by the significantly higher serum levels of TNFα, and PTX administration alleviated the low-grade inflammation associated with IR as indicated by the significant increase in serum adiponectin levels. This is in accord with our previous work showing that PTX alleviated low-grade inflammation in an IR animal model [26]. In addition to its role in suppressing low-grade inflammation, adiponectin is known to regulate glucose utilization through the activation of 5′-AMP-activated protein kinase (AMPK) [28], [29]. However, we did not find significant effect of PTX on serum glucose or insulin level as would be expected as a result of the increased serum adiponectin level. The effect on glucose utilization might need higher level of adiponectin than the level sufficient to suppress cardiac ischemia and dysfunction. In the study of Yamauchi et al [28], adiponectin start to improve glucose utilization at a dose of 10 µg/10 g body weight in mice, which is very high compared with the increase in adiponectin serum level by PTX seen in this study.

The possibility that alterations in the circulating lipid profile contributed to the IR-PTX cardiac dysfunction interaction was also investigated. The IR model used in this study did not show significant changes in, and PTX administration did not affect, the serum lipid parameters in normal or IR animals. These observations exclude the role of serum lipids in the IR-PTX cardiac dysfunction interaction.

## Conclusion

PTX alleviates cardiac diastolic ischemia and dysfunction associated with IR directly through mechanisms involving inhibition of the low-grade inflammation associated with IR.
